# Mechanisms of paeoniaceae action as an antidepressant

**DOI:** 10.3389/fphar.2022.934199

**Published:** 2023-02-08

**Authors:** Wanxu Guo, Xiaoxiao Yao, Ranji Cui, Wei Yang, Lei Wang

**Affiliations:** Jilin Provincial Key Laboratory on Molecular and Chemical Genetic, The Second Hospital of Jilin University, Changchun, China

**Keywords:** depression, paeoniflorin, antidepressant, neuroprotection, apoptosis, neuroinflammation

## Abstract

Paeoniflorin (PF) has been widely used for the treatment of depression in mice models, some Chinese herbal compound containing PF on treating depression, such as Xiaoyao San, Chaihu-Shugan-San, Danggui Shaoyao San etc. Many experiments are also verifying whether PF in these powders can be used as an effective component in the treatment of depression. Therefore, in this review the antidepressant effect of PF and its mechanism of action are outlined with particular focus on the following aspects: increasing the levels of monoamine neurotransmitters, inhibiting the HPA axis, promoting neuroprotection, enhancing neurogenesis in the hippocampus, and elevating levels of brain-derived neurotrophic factor (BDNF). This review may be helpful for the application of PF in the treatment of depression.

## Introduction

Depression is one of the most common psychiatric sick and is characterized by significant and persistent long-term low mood with suicide being its most serious consequence. Surveys reveal that the prevalence of depression was highest among younger adults aged 18–29 years; however, the prevalence of depression in people aged over 45 is low (Wittchen et al., 1994). Depression and has become a global disease burden and its prevalence continues to increase. Studies have shown that the prevalence of depression increased significantly from 1994 to 2014, and the prevalence rate from 2004 to 2014 was higher than that from 1994 to 2003 (Lim et al., 2018). Age has also become a key factor in the incidence of depression, the prevalence of depression is constantly growing, especially during adolescence (McCarron et al., 2021). A survey in 2020 showed that college students have a high prevalence of depression (Gao et al., 2020).

Currently, there are several antidepressant drugs in clinical use, including tricyclic antidepressants (TCAs); monoamine oxidase inhibitors (MAOIs) and selective norepinephrine reuptake inhibitors (SNRIs) (Qiu et al., 2013a). However, these antidepressants have serious side-effects such as drowsiness, dryness of mouth, headaches, nausea, and sexual dysfunction. Furthermore, chronic or acute administration of antidepressants may lead to weight gain and even obesity. In general, TCAs and MAOIs may be more likely to cause weight gain than SNRIs (Fava, 2000). However, when used excessively antidepressants may cause seizures or hepatotoxicity manifesting as a transient increase in liver enzymes ultimately causing fulminant hepatic failure (FHF) (Lucena et al., 2003; Judge and Rentmeester, 2013). A previous study revealed that the onset time of hepatotoxicity caused by antidepressants ranged from 5 days to 3 years (Park and Ishino, 2013). Therefore, the clinical response and efficacy of antidepressants need to be optimized, and the development of new drugs with low toxicity is urgently needed.

Paeoniflorin (PF) is a type of monoterpene glycoside, which is derived from the root of paeonia or peony and is extracted from Radix Paeoniae Alba, which is a traditional low toxic Chinese herbal medicine widely used in the treatment of depression. Some traditional Chinese medicines containing PF have been used in the treatment of depression, such as Xiaoyao San (Chen C et al., 2020), Chaihu-Shugan-San was also proved to have a therapeutic effect on depression as early as 2004 (Kim et al., 2005). Danggui Shaoyao San is also widely used to treat depression (Xu et al., 2011; Huang et al., 2012). The pharmacological mechanisms (such as anti-neoplastic, anti-inflammatory, anti-oxidant, anti-apoptotic, or neuroprotective) of PF have been reported (Zhao et al., 2013; Tao et al., 2016; Ouyang et al., 2017; Zhang and Wei, 2020). For example, PF plays an anti-tumor role by inhibiting the expression of S-phase kinase associated protein (SKP) two in glioma cells (Ouyang et al., 2017). PF can also reduce the expression of TLR4 and MyD88, as well as the phosphorylation of IκBα and NF-κB p65, which could alleviate atherosclerosis and chronic inflammation (Li et al., 2017). However, there has been increasing emphasis on the antidepressant effects of PF in recent years. PF has been shown to elicit antidepressant activity in all of the tested mouse and rat models including chronic unpredictable mild stress (CUMS), forced swim tests (FST), tail suspension tests (TST), medicinal induction, post-stroke depression, and menopause related depression (Wang X.-L. et al., 2021).

Many other studies have proved the antidepressant effect of PF. It has been demonstrated that PF exerts neuroprotective effects via the extracellular signal-regulated kinase–cyclic adenosine monophosphate response element binding protein (ERK-CREB) signaling pathway in a CUMS-induced hippocampal damage rat model (Zhong et al., 2019). Furthermore, in a rat model of post-stroke depression (PSD), PF treatment significantly increased the levels of phosphorylated CREB (p-CREB) and brain-derived neurotrophic factor (BDNF) in the CA1 region of the hippocampal complex (Hu et al., 2019).

It has been reported that there are a number of molecular mechanisms underlying the antidepressant effects of PF, such as the effects of monoaminergic neurotransmitters on depression and the effects of the hypothalamic–pituitary–adrenal (HPA) axis on depression. In this article we will review the recent research on the antidepressant effects of PF and discuss the mechanisms underlying these effects. It is expected that PF could be exploited by studying the underlying molecular mechanisms.

## Effects of monoamine neurotransmission on depression

An increasing number of studies have confirmed that monoamine neurotransmission, serotonin (5-HT), noradrenaline (NE), and dopamine (DA) are related to the neurophysiological process of depression. Physiological changes in abnormal 5-HT, NE, and DA signal transduction lead to changes in receptor regulation or function or impaired intracellular signal processing (Liu et al., 2018). Monoamine neurotransmitters have an extensive range of biological functions and are an important regulatory class for a series of physiological activities in the central nervous system (CNS, such as mental activity, behavioral states and emotion). Norepinephrine may be related to alertness, anxiety, attention, and attitude to life whereas 5-HT is involved in anxiety, and obsessive-compulsive disorders, and dopamine is involved in attention deficits, motivation, pleasure, and reward. Therefore, increasing any of these three neurotransmitters will elevate mood, but the other elements of depression may be particularly responsive to a certain neurotransmitter (Nutt, 2008). 5-HT in the brain has a significant role in mood alleviation, hunger, and sleep regulation. In addition, a healthy diet may also alleviate the symptoms of depression. One research has reported that fruits and vegetables are rich in 5-HT, which is influenced by the blood–brain barrier (BBB). Tryptophan is the precursor of 5-HT. 5-HT is synthesized through decarboxylation (Shabbir et al., 2013). The effect of NE in depression was also reported. NE is projected from the cell bodies in the locus coeruleus to the limbic system to regulate emotion, and NE transporter (NET) levels in the locus coeruleus are decreased in patients with depression who commit suicide. In addition, NET knockout mice exhibited increased extracellular NE levels and developed resistance to depression-like behaviors (Briley and Chantal, 2011). These results suggest that NE system enhancement could ameliorate depression-like behaviors in mice.

To date, the monoamine hypothesis does not address key issues, for example, drugs that enhance the delivery of 5-HT or norepinephrine are not necessarily effective in treating depression. Despite its limitations, the development of the monoamine hypothesis is of great importance for the understanding of depression and the development of novel, safe and effective drugs for its treatment (Hirschfeld, 2000).

Danzhi Xiaoyao San (DXS), a modified formula from Xiaoyao San, is a canonical Chinese medicine. This herbal formula is composed of the following herbs: Radix Bupleuri, Paeoniae Radix Alba, Angelica sinensis, bighead atractylodes rhizome, Poria cocos, bark of tree peony root, Fructus Gardeniae, Mentha haplocalyx, and licorice (Wu et al., 2016). DXS, which contains PF, has been combined with antidepressants in the clinic. It was shown that DXS exerts antidepressant-like effects in CUMS rats by inhibiting pro-inflammatory cytokine levels, inhibiting the activation of indoleamine 2,3-dioxygenase (IDO) in the hippocampus and upregulating the hippocampal contents of tryptophan and 5-HT (Zhu et al., 2015). Another study demonstrated that treatment with total glucosides of peony could inhibit monoamine oxidase activity to maintain the levels of monoamine neurotransmitters in the rat brain, while peony extract (containing 48.99% PF and 18.99% albiflorin) could increase the contents of 5-HT and NE in the brain of CUS-induced rats (Qiu et al., 2013b).

Moreover, experiments have been conducted to investigate the effects of PF in an animal model of depression in menopause. In rats which were given 10 mg/kg PF by gavage for 2 weeks, PF exerted antidepressant effects by upregulating 5-HT_1A_ receptor (5-HT_1A_R) mRNA expression and downregulating 5-HT_2A_ receptor (5-HT_2A_R) mRNA expression (Huang et al., 2015). PF can simultaneously exert antidepression effects via polypharmacology; for example, rats subjected to the forced swimming test (FST) exhibited decreased plasma and hippocampus 5-hydroxytryptamine, norepinephrine, and dopamine levels and reduced plasma BDNF and superoxide dismutase (SOD) levels (Mu et al., 2020).

Many studies have shown an antidepressant effect of PF, and its possible implication for monoamine neurotransmitters. The results show that PF can increase the levels of monoamine neurotransmitters in the mouse hippocampus (Qiu et al., 2013a). In another study, rats treated with the monoamine reuptake inhibitor reserpine received intragastric doses of peony glycosides of 40, 80, and 160 mg/kg. Total glucosides of peony (TGP) at 80 and 160 mg/kg significantly inhibited the activity of MAO-A and MAO-B (Mao et al., 2008). Monoamine oxidase inhibitors (MAOIs) can significantly alleviate depression by reducing the metabolism of monoamine neurotransmitters and also, preserve monoamine neurotransmitter concentration by inhibiting monoamine oxidase (MAO) which cause their breakdown. In addition, the activities of MAO-A and MAO-B were increased in CUS-induced rats. This phenomenon was reversed after treatment with TGP (whose major active component is PF) when TGP was given intragastrically 30 min before each stressor once every day for 24 days (Mao et al., 2009b). These findings suggest that the antidepressant effect of PF may be mediated through inhibition of MAO and therefore, has an antidepressant effect by increasing monoamine neurotransmitter levels.

**FIGURE 1:**
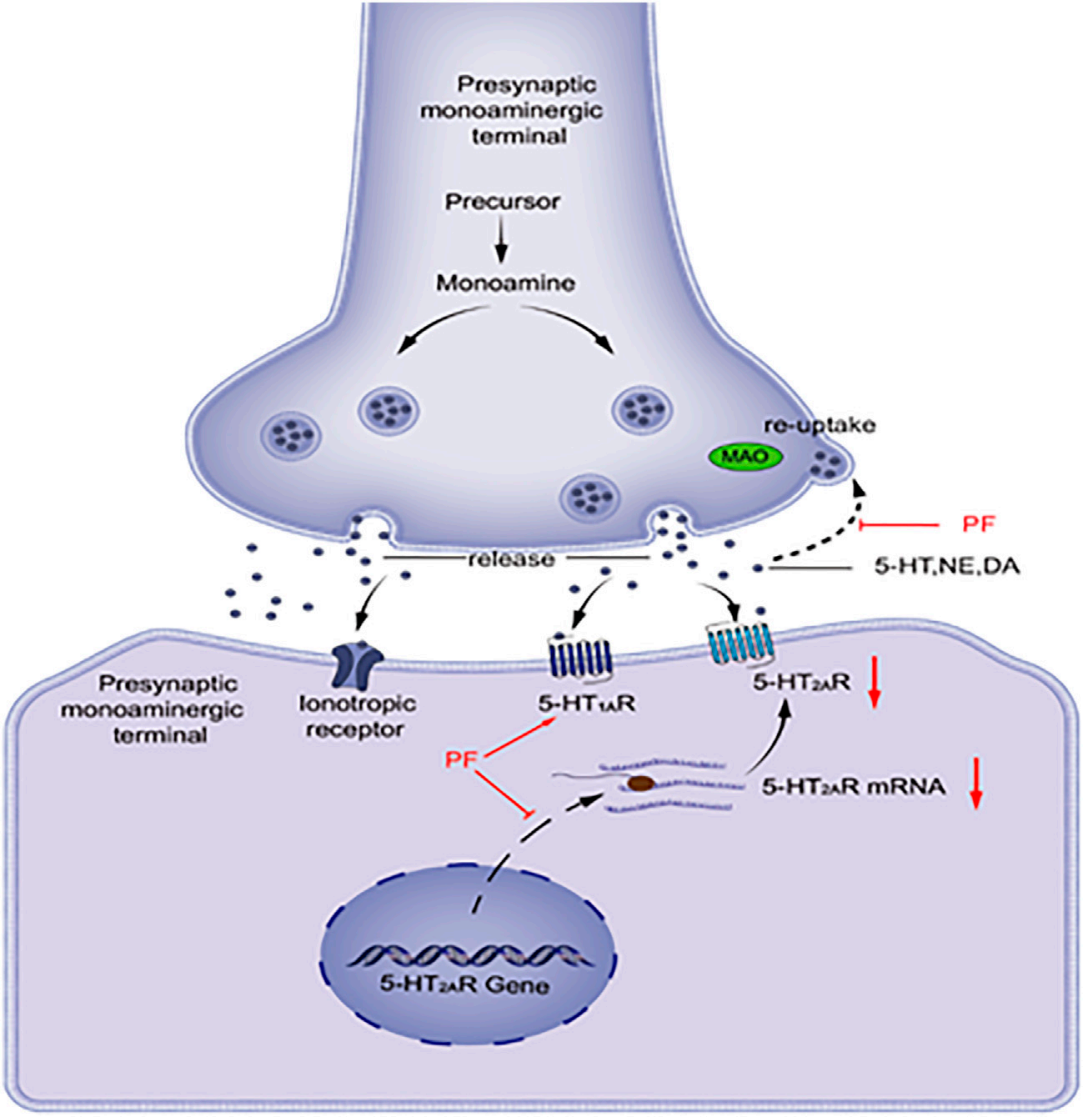
DA, dopamine; MAO, monoamine oxidase; NE, noradrenaline; PF, paeoniflorin; 5-HT, serotonin; 5-HT_1A_R, 5-HT_1A_ receptor; 5-HT2AR, 5-HT_2A_ receptor.

As shown in Figure 1, visual representation of the pathway by which monoamine neurotransmitters are released into and removed from the synaptic space. 5-HT, NE, and DA are synthesized and released into the synaptic cleft. These neurotransmitters can interact with receptors on postsynaptic cells or bind to reuptake channels and be oxidized by MAO. Patients with depression have lower levels of monoamine neurotransmitters, and PF can increase the levels of monoamine transmitters by inhibiting the activity of MAO. The 5-HT_1A_ and 5-HT_2A_ receptors functionally compete with each other. The 5-HT_1A_ receptor function decreases and the 5-HT_2A_ receptor function increases in patients with depression. PF can increase the mRNA and protein expression levels of 5-HT_1A_ receptors in the hypothalamus area in model rats, while the expression levels of 5-HT_2A_ receptors are decreased.

### Effect of the hypothalamic–pituitary–adrenal axis

The HPA axis is implicated in the pathophysiology of depression, which involves the brain, the pituitary gland, and the adrenal glands, which regulate glucocorticoid (GC) production. Cortisol, a major stress hormone released by the adrenal glands, shows high affinity with mineralocorticoid receptors (MRs) and low affinity with glucocorticoid receptors (GRs). MRs primarily function in the hippocampus, while GRs play feedback roles in the brain and the pituitary gland. We speculated that the pathogenesis of depression could be caused by imbalance of GR and MR regulation in the HPA system (Keller et al., 2017). Patients with severe affective disorder of depression usually have high cortisol levels. It was found that depressive symptoms were apparently relieved when cortisol levels were reduced in either way. Therefore, it seems that steroids themselves play an important role in the occurrence and delay of depression (Pearsonmurphy, 1991).

The HPA axis is the endocrine core of the stress response in mammals, and involves corticotropin-releasing hormone (CRH) which is secreted by the hypothalamus, causing the release of adrenocorticotropic hormone (ACTH) in the anterior pituitary gland, and GC or corticosterone (CORT) in the adrenal glands. Changes in the HPA axis has been consistently found in patients with depression (Fischer et al., 2017). Misalignment of the HPA axis is believed to be mainly driven by interruption of the GR-dependent negative feedback (i.e., GR resistance) (Troubat et al., 2021). However, the change in GR levels did not directly cause depression-like behavior. Hypercortisolemia and disturbances of GR function were not always observed in the clinical manifestations of depression. Corticotropin-releasing factor (CRF) levels were increased in patients with depression, according to the autopsy reports, while the changes in CRF levels might be the basis of the influence of GR manipulation (Neigh and Nemeroff, 2006).

In rats given 80 mg/kg or 160 mg/kg CUMS through intragastric gavage once a day for 6 weeks, TGP treatment reduced the immobility time in the FST (19% and 27%, respectively), decreased serum CORT levels in a dose-dependent manner (23–30), and increased the GR mRNA level. Thus, long-term administration of TGP could effectively relieve the CUMS-induced depressive-like symptoms. TGP might exert its antidepressant effects by changing the function of the HPA axis (Mao et al., 2009a). Experiments have shown that PF can reverse the activity of the HPA axis in depressed mice, and reduce the levels of CRH, ACTH and CORT (Mu et al., 2020). In rats treated with chronic unpredictable stress (CUS), PF treatment significantly decreased the levels of ACTH and CORT. Furthermore, PF treatment attenuated the CUS-induced reduction of norepinephrine, 5-HT or its metabolite, 5-hydroxyindoleacetic acid (Qiu et al., 2013b). These results indicate that the regulation of the HPA axis and the up-regulation of the 5-HT and noradrenergic systems are important mechanisms for the antidepressant-like effect of PF in CUS-treated rats (Lichun et al., 2020).

**FIGURE 2:**
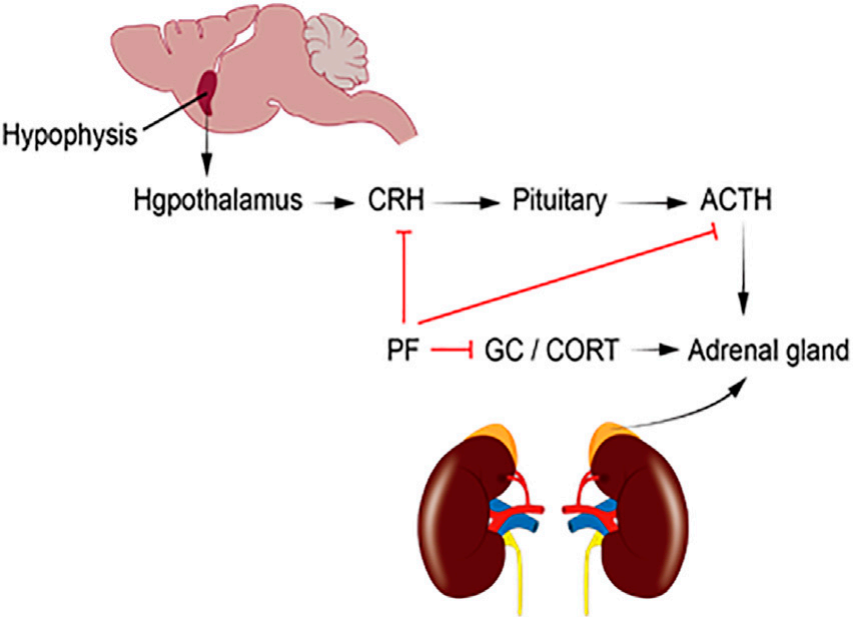
ACTH, adrenocorticotropic hormone; CORT, corticosterone; CRH, corticotropin-releasing hormone; HPA, hypothalamic–pituitary–adrenal; GC, glucocorticoid; PF, paeoniflorin.

As shown in Figure 2, depression patients with hyperactive HPA axis are mainly characterized by elevated CRH, ACTH, CORT, and metabolite levels in the central and peripheral nervous system, especially in the hypothalamus. PF can reduce the serum levels of CRH, CORT, and ACTH and restore the function of the HPA axis. This significantly improves the depression-like behavior of model rats.

## Neuroprotection

Preclinical studies have shown that chronic stress can cause changes in the number and shape of neurons and glial cells in brain regions related to mood disorders including the hippocampus (HP), prefrontal cortex (PFC), cingulate cortex, nucleus accumbens and amygdala (AMY). Brain imaging and autopsy studies have found that the extent of branching, complexity of the dendrites and the number of neurons and glial cells in these brain regions are reduced in patients with depression (Duman, 2009).

Specific experiments have also shown that the pathogenesis of antidepressant treatment is related to HP volume reduction and PFC dysfunction. Therefore, neuronal damage is also related to depression (Schoenfeld et al., 2017; Seo et al., 2017). Currently, many *in vitro* and *in vivo* experiments have shown that PF has a neuroprotective effect on various types of neuronal damage and next we will summarize the neuroprotective mechanisms associated with its treatment (See Table 1).

**TABLE 1:** *In vitro* studies on the neuroprotective effects of PF.

Pharmacological effects	Cell lines	Model drugs	PF doses	Duration (hours)	Cell and molecular changes	References
Inhibition of oxidative stress	PC12	CORT	1/10/50 μM	2	Cell viability ↑, ROS and MDA ↓, nerve growth factor mRNA ↑	Mao et al. (2012)
	PC12	6-OHDA	3/10/30 µM	2	Apoptosis ↓, GSH ↑, ROS/protein kinase Cδ/NF-κB ↓	Dong et al. (2015)
Balance between ion concentration and ion channel activity	PC12	NMDA	1/10/50 μM	2	Cell viability↑, intracellular Ca^2+^concentration↓, Calbindin-D28 K mRNA ↑, LDH release ↓	Mao et al. (2011b)
	Rat hippocampal neurons	KCl	50/100/200 μM	4	Intracellular Ca^2+^ concentration ↓	Song C et al. (2017)
Inhibition of apoptosis	PC12	MPP+	20/50/100/200/400 μM	3	Cell viability ↑, LDH release ↓, intracellular Ca^2+^ concentration and cleaved poly (ADP-ribose) polymerase ↓	Wang et al. (2013b)
	Rat hypothalamic neurons	Tributyltin chloride	25/50/100 μM	24	Neuronal apoptosis ↓, Bax/Bcl-2 ratio ↓, caspase-3 activity ↓, MKK4-c-JNK ↑, MMP ↑	Cong et al. (2019)
Inhibition of neuroinflammation	Rat hippocampal slices	Lipopolysaccharide	0.25/0.5/1/2 mM	0.5	Hippocampal cell death ↓, IL-1β and NO ↓ microglial cells the release of TNF-α, IL-1β, and NO↓	Zhou et al. (2019)

6-OHDA, 6-hydroxydopamine; GSH, glutathione; IL, interleukin; JNK, Jun N-terminal kinase; LDH, lactate dehydrogenase; MDA, malondialdehyde; MMP, mitochondrial membrane potential; MPP+, methyl-4-phenylpyridine ion; MKK4, mitogen-activated protein kinase kinase 4; NF-κB, nuclear factor κB; NMDA, N-methyl-d-aspartate; NO, nitric oxide; PF, paeoniflorin; PC12, pheochromocytoma; ROS, reactive oxygen species; TNF-α, tumor necrosis factor-α.

### Oxidative stress and depression

Oxidative stress is defined as an imbalance between reactive oxygen species (ROS) production and the antioxidant capacity of the cell. Oxidative stress is a major cause of depressive disorders (Bhatt et al., 2020). Excess production of ROS by the cells can cause damage to DNA, proteins, and lipids, and eventually lead to cell death (Black et al., 2015). Oxidative stress plays an important role in the pathophysiology of major depression through the actions of free radicals, non-free radical molecules, ROS and nitrogen-based molecules (Vaváková et al., 2015). As the brain is the major consumer of oxygen, oxidative stress can easily damage the CNS and accelerate the occurrence of mental illnesses, such as depression. Therefore, abnormal levels of oxidative stress markers are often seen in many depressive patients. DNA oxidation in Brodmann area 10 of the brain is significantly increased in depression (Szebeni et al., 2016) and it has been shown that 8-hydroxy-2′-deoxyguanosine (8-OHdG) and F2-isoprostanes (markers of DNA oxidation and lipid damage) act as reliable marker for increased oxidative stress (Black et al., 2015). A study found that 8-OHdG and F2-isoprostane levels were increased in patients with depression and suggested that oxidative stress increased and reduced the levels of antioxidants, indicating that depression is accompanied by increased oxidative damage (Black et al., 2015). High concentrations of glutamate can also induce neurotoxicity in rat pheochromocytoma cells (PC12) and oxidative stress may induce glutamate-induced neuronal damage. Whereas PF has a neuroprotective effect on PC12 cell neurotoxicity induced by glutamate, as it can inhibiting oxidative stress (Mao et al., 2010b).

Nerve growth factor (NGF) has powerful neuroprotective and nerve reparatory functions. One study reported that antidepressants could increase mRNA levels of NGF in CORT-induced PC12 cells, activate adenylate cyclase (AC) in the cell membrane, increase intracellular cAMP levels, and upregulate the cAMP-response element binding protein (cAMP-CREB) cascade and NGF expression (Yun-Feng et al., 2003). In CORT-treated PC12 cells (cell viability 41%), PF (1, 10, and 50 μM) can increase cell viability (to 49, 55, and 61%, respectively) and reduce intracellular ROS levels (to 169%, 149%, and 132% of the control value, respectively) and malondialdehyde (MDA) levels (to 180%, 161%, and 146% of the control value, respectively). PF can also reverse the decrease in NGF mRNA levels caused by CORT (51% of the control value) in PC12 cells, indicating that PF exerts its neuroprotective effect by inhibiting oxidative stress and up-regulating NGF expression (72%, 82%, and 87% of the control value, respectively) (Mao et al., 2012). PF can also inhibit mitochondrial 6-hydroxydopamine (6-OHDA)-induced apoptosis in PC12 cells by increasing the antioxidant capacity of glutathione (GSH), and can significantly attenuate 6-OHDA-induced nuclear factor κB (NF-κB) translocation and block the 6-OHDA-induced upregulation of protein kinase Cδ (PKCδ). These results indicate that the inhibition of apoptosis in PC12 cells caused by PF may be mediated through the inhibition of the ROS/PKCδ/NF-κB signaling pathway (Dong et al., 2015). The survival rate of CORT-induced PC12 cells decreased to 30%, and it significantly increased after TGP (1, 5, or 10 mg/L) treatment (to 38%, 45%, and 49%, respectively). Similarly, TGP (1, 5, or 10 mg/L) treatment decreased the intracellular ROS (160%, 148%, and 129% of the control value, respectively; CORT-treated 195%) and MDA (183%, 160%, and 145% of the control value, respectively; CORT-treated 214%) levels and increased the GSH (55%, 63%, and 71% of the control value, respectively; CORT-treated 42%) and SOD (52%, 57%, and 60% of the control value, respectively; CORT-treated 40%) levels. The level of catalase (CAT) (59% and 60% of the control value, respectively; CORT-treated 43%) also increased after TGP (5 and 10 mg/L) treatment. This suggested that TGP partially reversed the CORT-induced neurotoxicity (Mao et al., 2011a). These data strongly suggest that one of the mechanisms of neuronal protection produced by PF is the inhibition of oxidative stress.

### Balance between ion concentration and ion channel activity

Glial calcium (Ca^2+^) is involved in various forms of pathological processes in the nervous system (Nedergaard et al., 2010). Calcium pumps have high affinity for intracellular calcium ions and can stabilize the intracellular Ca^2+^ concentration. The calcium pump is the main factor reducing intracellular Ca^2+^ concentrations in hippocampal neurons (Mata and Sepúlveda, 2005). Disruption of mitochondrial dynamics can lead to calcium overload in cells and cause nerve cell damage or death (DeCoster, 1995). Ca^2+^ participates in the transmission of the depolarizing signal and contributes to synaptic activity. Ca^2+^ controls specific neuronal processes, such as long-term potentiation (LTP) or depression of synaptic transmission (Brini et al., 2014). Homeostatic control of intracellular Ca^2+^ levels plays an important role in the regulation of neuronal activity, including neurotransmitter release, synaptic plasticity, memory storage, and neuronal cell death (Calvo-Rodriguez et al., 2020). Glutamate activates postsynaptic receptors in excitotoxicity, including the ionotropic N-methyl-d-aspartate (NMDA) and 2-amino-3-(3-hydroxy-5-methylisoxazol-4-yl) proprionate (AMPA). Therefore, the excessive synaptic release of glutamate will lead to the imbalance of Ca^2+^ homeostasis, and eventually lead to apoptosis (Arundine and Tymianski, 2003). Elevated intracellular Ca^2+^ levels can activate a range of calcium-dependent signaling pathways (Takemoto-Kimura et al., 2017). Calmodulin (CaM) is expressed ubiquitously in eukaryotic cells and is involved in many key signaling pathways such as the control of growth, proliferation and motility, and changes in intracellular Ca^2+^ concentrations can regulate CaM (Chin and Means, 2000). Ca^2+^/calmodulin-dependent protein kinase II type (CaMK-II) is present in the brain and can increase Ca^2+^ levels through voltage-dependent channels and interact with CaM (Rothschild and Tombes, 2020) demonstrating that Ca^2+^, CaM, CaMK-II all play a key role in the nervous system.

Research has shown that abnormal serum calcium levels cause cognitive impairment in patients with depression and in the nervous system, NMDA receptors allow the influx of extracellular Ca^2+^ causing the activation of signaling pathways: calcineurin induces long-term depression (Grützner et al., 2018). Therefore, maintaining the correct regulation of calcium channels is one of the mechanisms that protect the nervous system. Calbindin-D28 K is one of the primary calcium-binding proteins in the brain and maintains the intracellular calcium balance by binding excessive Ca^2+^, which can protect normal activation and function of neurons and inhibit neuronal apoptosis induced by intracellular Ca^2+^ overload. Experiments showed that PF could reverse the mRNA level of Calbindin-D28 K in PC12 cells treated with glutamate and decrease the intracellular free calcium concentration (Shifman et al., 2006; Mao et al., 2010b).

### PF exerts neuroprotective effects by maintaining Ca^2+^ homeostasis and regulating Ca^2+^ signaling pathways

NMDA receptors are prevalent in the cerebral cortex, hippocampus, striatum, and amygdala. Importantly, damage to these regions can cause emotional and cognitive deficits. Activated NMDA receptors cause Ca^2+^ influx, and calcium overload could activate nitric oxide synthase (NOS), producing nitric oxide (NO) (Li et al., 2006). It has been found that PF has a protective effect on NMDA-induced neurotoxicity in rat PC12 cells as it can improve cell viability, reduce the release of lactate dehydrogenase (LDH), reverse the increase in intracellular Ca^2+^ concentrations and the decrease the mRNA levels of D28 K (calcium-binding protein) (Mao et al., 2011b). These results indicate that PF exerts a neuroprotective effect on NMDA-induced PC12 cell neurotoxicity at least partially through Ca^2+^ antagonism. Lots of experiments demonstrated that PF suppressed intracellular Ca^2+^ overload and the expression of CaMKII (Zhong et al., 2009; Wang et al., 2013a; Zhang et al., 2017). In a premenstrual syndrome (PMS) depression model, PF could restore the phosphorylation level of CaMKII in Ca_v_1.2-induced CaM/CaMKII signaling pathway. In addition, experiments have shown that PF can significantly inhibit the increase in intracellular Ca^2+^ concentration and Ca_v_1.2 current density induced by KCl, may regulate the CaM/CaMKII pathway through the regulation of Ca_v_1.2 (Song C et al., 2017).

### Apoptosis and depression

Dysregulation of apoptosis can damage neuroblasts, and ultimately lead to neurodegenerative diseases (Ghavami et al., 2014), suggesting a very important role for neuronal apoptosis in many CNS disorders. Diabetes-related depression rats exhibit hippocampal neuronal apoptosis by the aberrant Glu–GluR2–Parkin pathway, which is also responsible for depressive-like behaviors and monoamine neurotransmitter deficiency in rats (Liu et al., 2021). Stress also results in apoptosis (Lucassen et al., 2006). As is well known, apoptosis is controlled by pro-apoptotic family members (such as Bax) and anti-apoptotic family members (such as Bcl-2). A study has shown that a combination of isolation and CUMS stimulation after cerebral ischemia significantly upregulated neuronal apoptosis and the expression levels of pro-apoptotic proteins in the hippocampus (Wang A. et al., 2021). MicroRNAs (miRNAs) and connective tissue growth factor (CTGF) are also involved in hippocampal neuronal apoptosis in CUMS-induced depression-like mice (Pei et al., 2020). Prolactin receptor (PRLR), an antidepressant factor, participates in depression by the JAK2–STAT5 signaling pathway. Additionally, increased numbers of apoptotic cells and necrotic cells were observed in chronic mild stress (CMS)-induced depression mice, and the expression of caspase-3, Bax, and proteins involved in the JAK2–STAT5 signaling pathway was decreased after PRLR silencing, along with increased expression of BDNF and Bcl-2 (Tian et al., 2019).

Many studies have shown that the protective effect of PF is related to the anti-apoptotic pathway. As a potent neurotoxin, methyl-4-phenylpyridine ions (MPP^+^) can induce neuronal apoptosis and neurodegeneration. For example, PF (the most significant effect was observed at a dose of 200 μM) improves the viability of PC12 cells differentiated in MPP^+^ and also inhibits excessive release of LDH. It can also protect cells from apoptosis induced by DNA damage and reverse the concentration of MPP^+^ on B cell lymphoma and inhibits the increase of ADP-ribose polymerase which is involved in apoptosis (Wang et al., 2013b). Moreover, studies have shown that PF could protect neurons against oxidative damage and the role of mitophagy in apoptosis (Mayer and Oberbauer, 2003; Haeberlein, 2004; Cao et al., 2010). In addition, PF can inhibit the mitogen-activated protein kinase 4- c-Jun N-terminal kinase (MKK4-JNK) signaling pathway, down-regulate the level of cleaved-caspase-3 and up-regulate the ratio of Bcl-2/Bax, and significantly protect hypothalamic neurons from TBTC-induced cytotoxicity, apoptosis and the reduction in MMP (Cong et al., 2019). Further, PF also inhibits neuronal apoptosis by many pathways, such as by activating the Nrf2/ARE pathway [73] [74]. The survival rate of PC12 cells was 37% when the CORT concentration was 200 μM, and the survival rate of PC12 cells increased gradually (45–55%) after treatment with TGP. TGP could also reduce caspase-3 activity induced by CORT and upregulate the Bcl-2/Bax ratio. All of these results indicate that TGP inhibits apoptosis by suppressing the mitochondrial apoptosis pathway (Mao et al., 2009c). These data suggest that the regulation of apoptosis-related proteins and their signaling pathways is the key to the anti-apoptotic effect of PF.

### Neuroinflammation and depression

An increasing number of studies have shown that immune cells are involved in the neurodegenerative response of the CNS and that neuroinflammation has been associated with many neurodegenerative diseases. Furthermore, Microglia and astrocytes have been found to trigger inflammatory processes in neurodegenerative diseases (Ising and Heneka, 2018). Neuroinflammation caused by stress could impair adult hippocampal neurogenesis and result in cognitive deficits. Stress could also activate microglia (Wu et al., 2021). Depression can lead to characteristic glial cell changes and increase the levels of pro-inflammatory cytokines. These are crucial factors in the event of neuroinflammation in the brain (Benatti et al., 2016). Induction of inflammation was also accompanied by changes in behavior and emotional changes, including depressive-like behaviors. These are closely related to the brain immune system and neuroinflammation (Nettis and Pariante, 2020). A study revealed that the levels of interleukin-6 (IL-6), tumor necrosis factor-alpha (TNF-α), and interleukin-8 (IL-8) were increased in cerebrospinal fluid (CSF) of patients with depression (Enache et al., 2019). An increase of pro-inflammatory cytokines levels can activate the NF-κB signaling pathway, which could upregulate expression of inflammatory genes, such as *cyclooxygenase-2* (*COX-2*). COX-2 produces prostaglandin, especially prostaglandin E2 (PGE2), which can enhance complex inflammatory cascades (Pitsillou et al., 2020). Increasing evidence demonstrates that neuroinflammation is inextricably linked with depression; thus, inhibiting neuroinflammation is also becoming a critical therapeutic strategy for depression treatment.

Fibroblast growth factor 2 (FGF-2) can regulate neuronal proliferation and differentiation and is involved in treatment outcomes of the antidepressant fluoxetine (Simard et al., 2018). A study revealed that lipopolysaccharide (LPS)-induced neuroinflammation decreases the level of FGF-2 (Chen M et al., 2020). PF (20, 40, or 80 mg/kg) given daily by gavage for a week prevented the decrease in FGF-2 levels in LPS-injected mice. PF also reversed the increased levels of TLR4, NF-κB phosphorylation, and NLRP3 in the hippocampus and the increased levels of the pro-inflammatory cytokines IL-6, IL-8, and TNF-α. PF also reversed the increased Iba1-labeled microglia and COX-2 levels. PF reduced the release of pro-inflammatory cytokines through binding with TLR4/NF-κB/NLRP3 and alleviated neuronal injury by cytokines (Cheng et al., 2021). In some neuroinflammatory models, the anti-inflammatory effect of PF on the nervous system has been confirmed where it has been shown to inhibit the excessive activation of astrocytes and microglia induced by arterial occlusion (MCAO) and prevent the production of pro-inflammatory mediators. PF treatment can prevent TNFα-induced apoptosis (Guo et al., 2012). Apoptosis signal-regulated kinase 1 (ASK1) can activate p38, JNK and MKK, and induce apoptosis and inflammation (Liles et al., 2018). PF has also been found to mimic the ASK1 inhibitor NQDI1 and inhibit ASK1 phosphorylation thereby reducing astrocyte and microglia responses and lowering the expression of inflammatory factors such as IL-1β and TNF-α. In LPS-treated hippocampal slices, PF reversed hippocampal cell death and reduced NO and interleukin (IL)-1β production, while also reducing the release of pro-inflammatory factors such as tumor necrosis factor-α and IL-1β, thereby preventing nerve damage caused by inflammation (Zhou et al., 2019).

## Hippocampal neurogenesis and brain-derived neurotrophic factor

Brain-derived neurotrophic factor (BDNF) is derived from neuronal cells in the brain. BDNF synthesis takes place in the areas involved in emotional and cognitive functions, such as the hippocampus and frontal areas (Phillips, 2017). BDNF reduction was also found in hippocampus of patients with depression (Kim et al., 2007; Birkenhäger et al., 2012). CUS and depressive-like symptoms are associated with a reduction in BDNF levels in the hippocampus and frontal cortex and decreased tropomycin receptor kinase B (TrkB) activity (Russo-Neustadt et al., 2001; Zhang et al., 2014). It was found that antidepressants enhanced BDNF levels in the hippocampus (Duman and Monteggia, 2006), neurogenesis (Caviedes et al., 2017), and the viability of hippocampal cells (Bergami et al., 2008). Antidepressants increase hippocampal neurogenesis (Hill et al., 2015) and studies have shown that serum- and glucocorticoid-inducible kinase 1 (SGK1) was related to depression, which is a mediator of the influence of cortisol on neurogenesis and glucocorticoid receptor (GR) function. It has also been shown that the level of SGK1 in the peripheral blood of patients with depression increases, and CORT reduces neuronal differentiation through SGK1-dependent mechanisms (Anacker et al., 2013). BDNF is a growth factor that affects the proliferation, differentiation, survival, and death of both neuronal and non-neuronal cells and also can control emotional and cognitive function. The level of BDNF in the neurons of patients with depression is very low. However, the expression of BDNF increases after taking antidepressants (Song M et al., 2017) suggesting that the deficiency of BDNF has an important impact on the physiology and pathology of patients with depression.

### PF exerts a significant antidepressant effect by regulating hippocampal neurogenesis and BDNF levels

Long-term treatment with TGP (80 and 160 mg/kg, intragastrically) can reverse CUMS-induced depressive-like behavior in rats. PF increased the BDNF protein and mRNA levels in the hippocampus and frontal cortex of rats exposed to CUMS (Mao et al., 2010a). PF can reverse depression-like behaviors induced by CUMS and PF treatment prevents the reduction in dendritic spine density the expression of BDNF and postsynaptic density protein 95 (PSD95) in the hippocampus of these mice (Liu et al., 2019). In the acute depression animal model caused by forced swimming in rats, PF significantly increased rat serum and hippocampal levels of BDNF, and has a protective effect on hippocampal pathomorphology (Mu et al., 2020). PF can significantly increase the expression of hippocampal complex CA1 and phosphorylated CREB (p-CREB) in post-stroke depression (Hu et al., 2019) and significantly increases the rate of sucrose consumption and 5-bromo-2-deoxyuridine-positive cells in the dentate gyrus of rats induced by CUMS. In addition to the enhanced protein expression and gene transcription of BDNF, PF can also activate the expression of tropomyosin receptor kinase B (high-affinity receptor for BDNF) (Chen et al., 2019) and importantly can promote hippocampal neurogenesis and up-regulate BDNF levels and thus help to relieve the symptoms of depression (see Table 2).

**TABLE 2:** Summary of the anti-depressive mechanisms of paeoniflorin.

Pharmacological effects	Models	PF doses (mg/kg)	Duration (day)	Molecular changes	References
Increase in monoamine neurotransmission	FST	50/100/200	7	Brain 5-HT, NE, and DA ↑	Qiu et al. (2013a)
Inhibition of the hypothalamic–pituitary–adrenal axis hyperfunction	FST	10	7	Plasma and hippocampus CRH, ACTH, and CORT ↓, 5- HT, NE, DA ↑	Mu et al. (2020)
	CUMS	30/60	28	Serum ACTH and CORT ↓ Brain 5-HT, NE, and 5-HIAA ↑	Qiu et al. (2013b)
Promotion of hippocampal neurogenesis and upregulation of brain-derived neurotrophic factor	CUMS	20	29	Hippocampal CA1 long-term potentiation, dendritic spine density, BDNF and PSD95 ↑	Liu et al. (2019)
	FST	30	1	*Hippocampus* and serum BDNF and TrkB ↑	Mu et al. (2020)
	CUMS	60	28	Hippocampal BDNF proteins and mRNA, and TrkB ↑	Chen et al. (2019)

5-HIAA, 5-Hydroxyindole-3-acetic acid; 5-HT, 5-hydroxytryptamine; ACTH, adrenocorticotropic hormone; BDNF, brain-derived neurotrophic factor; CORT, cortical; CRH, corticotropin-releasing ho€rmone; CUMS, chronic unpredictable mild stress; DA, dopamine; FST, forced swimming test; NE, norepinephrine; PF, paeoniflorin; PSD95, postsynaptic density protein 95; TrkB, tropomyosin receptor kinase B.

## Conclusion

To sum up, we mainly discussed two commonly used models of PF antidepressant. In the depression model, PF significantly improved the depression like behavior of mice, such as reducing the immobility time of FST and TST. In the neuron injury model, PF protected neuron damage caused by different toxins by inhibiting oxidative stress, neuronal apoptosis and modulation of ion channel etc.

Although many experiments have confirmed an antidepressant effect of PF, there are also a few questions. For example, PF is a hydrophilic compound with low fat solubility, and it has difficulty crossing the blood–brain barrier (Yang et al., 2018). Therefore, more experiments are needed in the future to find new neuroprotective effects of PF, explore new ways for PF to smoothly pass the blood–brain barrier, to improve the antidepressant effect of PF, and to optimize treatment regimens. It is also necessary to seek the optimal concentration and dose of PF for depression. Most importantly, PF can be used as a very effective treatment target for depression. We hope to make up for the deficiencies through experiments and apply PF to the clinical treatment of depression as soon as possible.
